# Impact of Ear Stage Drought Stress on Yield and Rhizosphere Metagenomic Profiles in Maize Cultivars with Contrasting Drought Tolerance

**DOI:** 10.3390/metabo16070493

**Published:** 2026-07-13

**Authors:** Qi Lu, Hongqun Zhao, Kureshi RuKeye, Yao Geng, Jincheng Du, Liyang Chen, Qiuhui Zhu, Congfang Xi, Jianbin Li

**Affiliations:** College of Agriculture and Biotechnology, Yunnan Agricultural University, Kunming 650201, China; 2023210153@stu.ynau.edu.cn (Q.L.); 2023210154@stu.ynau.edu.cn (H.Z.); 2022310177@stu.ynau.edu.cn (K.R.); 2024240243@stu.ynau.edu.cn (Y.G.); 2024210176@stu.ynau.edu.cn (J.D.); 2024210193@stu.ynau.edu.cn (L.C.); 15096639135@163.com (Q.Z.); 1992009@ynau.edu.cn (J.L.)

**Keywords:** maize, yield, rhizosphere microorganisms, drought stress, grain-roughening stage drought

## Abstract

**Background/Objectives:** Drought stress is a primary constraint on maize productivity, yet the role of rhizosphere microbial communities in modulating cultivar-specific drought resilience remains poorly understood. This study aimed to investigate the physiological and microbiome-mediated responses underlying differences in drought tolerance between contrasting cultivars to better understand drought tolerance mechanisms. **Methods:** Two maize cultivars with contrasting drought tolerance—NK718 (tolerant) and Zhongdan 808 (sensitive)—were subjected to drought stress at the V12 stage. We assessed yield components, oxidative stress indicators (Malondialdehyde (MDA)), and antioxidant enzyme activities (Superoxide Dismutase (SOD), Peroxidase (POD), Catalase (CAT)). Metagenomic sequencing was employed to analyze structural and functional shifts in the rhizosphere microbiota. **Results:** Drought significantly suppressed yield and physiological performance in both cultivars. However, the sensitive cultivar suffered more pronounced yield losses and severe oxidative stress, indicated by elevated Malondialdehyde (MDA) and decreased antioxidant enzyme activities. Conversely, the tolerant cultivar maintained superior physiological homeostasis. Metagenomic sequencing revealed drought-induced microbial shifts, including decreased Proteobacteria and Ascomycota, alongside increased Actinobacteriota and Mucoromycota. Notably, the drought-tolerant cultivar exhibited enhanced microbial community stability and more complex co-occurrence networks. Furthermore, it enriched specific functional pathways, such as phenylpropanoid biosynthesis, which positively correlated with yield stability and antioxidant capacity. **Conclusions:** Maize drought tolerance is underpinned by the coordinated regulation of plant physiological adaptation and the structural and functional stabilization of the rhizosphere microbiome. These findings offer a theoretical framework for developing breeding strategies that leverage root-microbe interactions to optimize maize yields under water-limited conditions.

## 1. Introduction

Maize (*Zea mays* L.) is China’s primary grain crop, and its stable yield is directly linked to national food security. In recent years, global climate change has led to a high frequency of extreme drought events, which have become the most critical abiotic stress factor limiting maize production [1].

Water deficit encountered by maize at different growth stages disrupts normal physiological metabolism, resulting in growth inhibition and ultimately a sharp decline in grain yield [2,3].The ear stage (from the twelfth leaf stage to the tasseling stage) is the critical water-demand period for maize and is extremely sensitive to water stress. Drought stress during this stage significantly hinders spikelet differentiation in the female ears and impairs pollination and fertilization. This, in turn, increases the bald tip length, reduces the kernels per spike and hundred-grain weight, serving as the pivotal cause of yield loss [4,5]. Therefore, grain yield and its direct components are widely recognized as the most reliable and direct indicators for phenotyping and evaluating drought resistance in maize varieties [6,7]. Drought stress leads to increased generation of reactive oxygen species (ROS) in plants, causing cellular oxidative damage. Plants can scavenge ROS to maintain redox homeostasis through antioxidant enzyme systems such as Superoxide Dismutase (SOD), Peroxidase, (POD) and Catalase (CAT). Meanwhile, Malondialdehyde (MDA) levels characterize the degree of membrane lipid peroxidation damage, and Proline (Pro) enhances plant drought adaptation through mechanisms such as osmotic adjustment and free radical scavenging [8]. Studies have shown that the elevation of SOD, POD, and CAT activities, along with the increase in Pro content and alterations in MDA content, collectively constitute the core physiological and biochemical indicator system for evaluating crop drought resistance [9]. However, plant drought resistance depends not only on endogenous physiological and genetic characteristics, but the micro-ecosystem governing plant–soil-microbe interactions also plays an irreplaceable role [10]. Drought stress not only directly alters soil physicochemical properties, but also fundamentally reshapes the diversity, composition, structure, and ecological functions of the rhizosphere microbial community [11].

Centering on these issues, extensive research has been conducted by domestic and international scholars from the perspectives of yield traits and the rhizosphere micro-ecosystem. Regarding yield traits, Cairns et al. (2013) found that combined high-temperature and drought stress during the flowering stage significantly increased the maize kernel abortion rate and decreased overall grain yield [12]. Hussain et al. (2019) further confirmed in controlled pot experiments that under concurrent heat and drought stress, key ear traits—such as ear length, ear diameter, and the number of grains per row—were all significantly reduced [13]. In terms of rhizosphere microbial responses to drought, important progress has also been made across various crops. Research has demonstrated that drought treatment significantly alters the rhizosphere microbiome structure, enriching specific stress-adaptive taxa such as Pseudomonadota (formerly Proteobacteria, including *Pseudomonas* species) and Bacteroidota to help maintain plant health [14]. Using the drought-tolerant potato genotype C93 and the drought-sensitive genotype Favorita as materials, Qin et al. (2025) revealed that the response patterns of rhizosphere microbial communities differed significantly among genotypes under drought conditions [15]. Similarly, host genetics strongly modulate the recruitment of stress-alleviating microbes. Studies on diverse grass lineages have demonstrated that, compared to drought-sensitive counterparts, drought-tolerant genotypes selectively enrich dominant bacterial phyla with stress-tolerance-enhancing functions in their rhizosphere, particularly Actinomycetota and Chloroflexota [16]. Research on citrus also indicated that drought significantly altered the rhizosphere microbial community composition, reducing the rhizosphere bacterial Shannon diversity of drought-tolerant cultivars by approximately 15%, while drought-tolerant cultivars also exhibited a more stable and complex bacterial network [17]. Furthermore, the impact of drought on bacterial beta diversity was prominent, especially in plant species with poor drought tolerance [7]. Xiang et al. (2025) revealed that drought stress shifts the microbial composition of the wheat phyllosphere, rhizosphere, and root endosphere by favouring Actinobacteriota and Ascomycota while depleting Pseudomonadota and Basidiomycota [18]. In recent years, the wide application of metagenomic sequencing technology has enabled researchers to systematically reveal the community remodeling patterns of rhizosphere microorganisms at the functional gene level and further characterize the symbiotic and competitive relationships among species through co-occurrence network analysis [19]. As for maize, studies have demonstrated that the maize rhizosphere is primarily enriched with Proteobacteria, Bacteroidetes, and Actinobacteria, whose dynamic succession is generally governed by one or two dominant taxa. In the early growth stage, copiotrophic genera such as Massilia (mostly belonging to Proteobacteria) predominate, whereas oligotrophic genera gain dominance in the later stage [20]. Different maize varieties vary in drought resistance: NK718 are drought-tolerant varieties, while Zhongnong 8B, Zi 330, and Zhongdan 808 are drought-sensitive varieties, among which NK718 possesses the highest richness of endophytic bacterial species [21,22,23].

Although the aforementioned studies have revealed the extensive impacts of drought stress on the rhizosphere microbial communities of various crops and preliminarily clarified the succession patterns of maize rhizosphere bacteria as well as the differences in drought resistance among varieties, systematic research focusing on the succession patterns of rhizosphere microbial communities in different drought-resistant maize varieties under drought stress at the ear stage remains relatively weak. The differences in rhizosphere microbial community structures among varieties with varying drought resistance under ear-stage drought stress, as well as their correlation mechanisms with yield and its components, remain unclear. More importantly, the multi-dimensional interaction mechanisms among microbial community remodeling, maize yield phenotypes, and antioxidant physiological indicators still lack systematic dissection. To this end, this study implemented drought stress during the ear stage of maize. In-depth investigations were conducted across multiple dimensions, including the species composition, diversity, co-occurrence network stability, and functional pathway characteristics of the rhizosphere bacterial and fungal communities, as well as their correlation analyses with plant physiological indicators and yield traits. The novelty of this study lies in the integration of macro-level agronomic and physiological drought—tolerant phenotypes with macro-level agronomic and physiological drought-tolerant phenotypes with micro-level rhizosphere ecological networks and functional predictions. This approach systematically reveals the response patterns of different varieties under drought stress, aiming to provide a new perspective for elucidating the “maize-rhizosphere microbe interaction” stress-resistance mechanism, and to offer a theoretical basis for promoting stable maize yields under drought conditions through micro-ecological regulation.

## 2. Materials and Methods

### 2.1. Experimental Materials

In this study, the maize hybrid varieties Zhongdan 808 (drought-sensitive variety, 01) [22] and NK718 (drought-tolerant variety, 03) [21] were selected as experimental materials.

Zhongdan 808 is a tall, large-eared, high-yielding, and excellent potential for both intercropping and silage. NK718 is a compact maize variety characterized by its high planting density tolerance, strong lodging resistance, fast grain dehydration, and excellent suitability for direct mechanical harvesting.

### 2.2. Location of the Experimental Site

The trial site is situated at the practical teaching base within the campus of Yunnan Agricultural University in Panlong District, Kunming City, Yunnan Province (25°14′ N, 102°75′ E, altitude 1995 m). From April to September 2025, Panlong District in Kunming City exhibited distinct dry and wet seasons. The overall climate was mild with excellent air quality and an absence of extreme heat. Specifically, April was dry with scarce rainfall, marking the end of the dry season; the rainy season began in mid-May, bringing a gradual increase in precipitation; June through August served as the main flood season, characterized by significantly heavier rainfall and frequent severe convective weather, with precipitation peaking in July; by September, the rainy season subsided, leading to reduced rainfall and a transition toward a drier and crisper climate.

The drought tolerance trials were conducted in a smart multi-span film greenhouse equipped with fully automated sprinkler and drip irrigation systems, as well as temperature control systems. The soil inside the greenhouse is dryland red soil, with Chinese cabbage as the previous crop.

### 2.3. Experimental Design

This study was conducted within a smart greenhouse, which was divided into two separate areas—one for well-watered (normal irrigation) control and one for drought treatment—using a brick-and-concrete partition (50 cm deep, 100 cm wide). The two areas were completely isolated from one another, with each area planted with the two maize varieties mentioned above. Four treatments were established: Variety 01 well-watered (JN01), Variety 01 under drought conditions (JD01), Variety 03 well-watered (JN03), and Variety 03 under drought conditions (JD03). A randomized complete block design was employed in the smart greenhouse, with three replicates, resulting in a total of 12 plots. Each plot covered an area of 14.4 m^2^, with guard rows of 1.2 m on all sides. Due to objective constraints such as the greenhouse layout, drought and water control conditions, and experimental management difficulties, three biological replicates were set as the standard and reasonable number of replicates for these microbiological trials.

For both the drought-stress and control plots, sprinkler irrigation was applied once after sowing and once during the seedling stage. Upon entering the jointing stage, drip irrigation was applied once (on 1 June), resulting in a cumulative irrigation volume of 38.7 m^3^ for all treatments. From the ear development stage to maturity, the control plots received four additional drip irrigations with a cumulative volume of 47.0 m^3^, whereas irrigation in the drought-stress plots was withheld starting from the late whorl stage.

### 2.4. Sowing and Field Management

Land preparation and sowing: On 15 April 2025, the land was tilled using a micro-tiller and rotary tiller, followed by soil leveling. Planting holes (approximately 30 cm deep) were dug using a wide-narrow row spacing pattern of (80 cm + 40 cm) × 50 cm. Sowing took place on 23 April, with three seeds sown per hole. Thinning was carried out at the three-leaf stage, leaving two plants per hole.

Fertilizer and water management: Basal fertilizer application comprised 3700 kg/hm^2^ of organic fertilizer and 570 kg/hm^2^ of compound fertilizer (N-P-K = 15:15:15). Prior to the drought treatment, the volumetric soil water content at a depth of 7.5 cm was maintained at approximately 21%. Following the imposition of drought stress, the soil water content in the control plots was maintained at around 13%, while the moisture levels in the drought-stress plots are detailed in Table 1.

Temperature control: The temperature in the experimental plots within the greenhouse was automatically regulated according to preset maximum and minimum temperatures (Table 2). When the temperature exceeded the maximum, fans and the cooling pad system were automatically activated to lower the temperature; when it fell below the minimum, the external ventilation windows were automatically closed to retain heat and raise the temperature.

### 2.5. Measurement of Indicators and Methods

#### 2.5.1. Yield and Related Traits

Five maize plants were randomly sampled consecutively from each replicate to determine the effective panicle number (EPN), biomass yield (BY), and economic yield (EY).

After drying the ears, the following parameters were measured: ear length (EL), ear diameter (ED), grain weight per ear (GW), kernels per ear (KPS), cob weight (CW), cob diameter (TR), barren tip length (BTL), number of rows per ear (NRE), number of grains per row (NGR), 100-kernel weight (HGW), and moisture content. Grain moisture content was measured using a grain moisture analyzer.

Ear length and barren tip length were measured using a ruler, estimated to one decimal place, accurate to 0.01 cm.

Ear diameter and cob diameter were measured using a vernier caliper, accurate to 0.01 mm.

The weight of stalk, cob, ear, and 100-kernel weight were weighed using an electronic balance, accurate to 0.01 g.

The formula for calculating economic yield (t/hm^2^) is as follows:

$$EY(t/{hm}^{2})=\frac{EPN\times KPS\times HGW}{100\times 1000\times 1000}$$

The formula for calculating biological yield (t/hm^2^) is: Stalk weight (t/hm^2^) plus economic yield (t/hm^2^).

#### 2.5.2. Physiological and Biochemical Traits

Samples of the ear leaves from 12 plots of each of the 4 treatments were collected successively at the stage of the large ear bract of corn (period A), when the corn leaves turned white and curled under drought treatment (period B), and at the harvest period of corn (period C). The samples were ground in liquid nitrogen in the laboratory and stored at −80 °C in a refrigerator as the original samples. The contents of MDA and Pro, as well as the activities of SOD, POD and CAT, were determined.

(1) Determination of Proline (Pro) Content.

The proline content was determined using the Personalbio G0111F Proline Assay Kit (Grace Biotechnology, Suzhou, China). A 0.1 g sample was weighed from the previously mentioned ultra-low temperature preserved raw maize leaf samples. 1 mL of the extraction buffer provided in the kit was added, and the sample was thoroughly homogenized in an ice bath. The homogenate was transferred to a 1.5 mL EP tube and extracted in a 90 °C water bath with shaking for 10 min. After cooling, it was centrifuged at 12,000 rpm and 25 °C for 10 min, and the supernatant was collected for testing. Next, 300 μL of the sample supernatant, 300 μL of glacial acetic acid, and 600 μL of Reagent I from the kit were sequentially added to the test tube. For the blank tube, the sample was replaced with 300 μL of distilled water, while the amounts of the other reagents remained identical to those in the test tube. All EP tubes were sealed, heated in a 95 °C water bath for 30 min, and then cooled to room temperature. 800 μL of the reaction mixture was transferred into a glass cuvette with a 1 cm optical path. The absorbance was measured at a wavelength of 520 nm using a spectrophotometer, and ΔA was calculated as Atext-Ablank. Based on the standard curve $y=0.1307x-0.013,(R^{2}=0.9999)$, the proline content was calculated using the following formula:

Pro Content (μg/g fresh weight)=[(ΔA+0.0071)÷0.1304]÷[W×V1÷V]×D=25.6×(ΔA+0.0071)÷W×D.

(V: Total volume of the extraction buffer (1 mL); V1: Volume of the extraction buffer added to the reaction system (0.3 mL); V2: Liquid sample volume (0.1 mL); W: Sample mass (g); D: Dilution factor).

(2) Determination of Malondialdehyde (MDA) Content.

The MDA content was determined using the Personalbio G0109F MDA Assay Kit (Grace Biotechnology, Suzhou, China). Similarly, a 0.1 g maize leaf sample was weighed, mixed with 1 mL of the kit’s extraction buffer, and homogenized in an ice bath. The mixture was centrifuged at 12,000 rpm at 4 °C for 10 min, and the supernatant was kept on ice for testing. 600 μL of the kit’s working solution and 400 μL of the test sample were added to an EP tube. After thorough mixing, the tube was incubated in a 90–95 °C water bath for 30 min. It was then removed, cooled in an ice bath, and centrifuged at 12,000 rpm at 25 °C for 10 min.The entire supernatant was transferred to a cuvette with a 1 cm optical path. Absorbance values were measured at wavelengths of 532 nm and 600 nm using a spectrophotometer. The content was calculated according to the following formula:

$$\begin{array}{r} MDA\;Content\;(nmol/g\;fresh\;weight)=[\Delta A÷(\varepsilon \times d)\times V2\times 10^{9}]÷\\ (W\times V1÷V)=16.1\times \Delta A÷W. \end{array}$$

(V: Total volume of the sample extraction buffer (1 mL); V1: Volume of the sample added to the reaction system (0.4 mL); V2: Total reaction liquid volume of the sample extraction buffer and working solution (1 × 10^−3^ L); d: Cuvette optical path length (1 cm); ε: MDA molar extinction coefficient (155 × 10^3^ L/mol/cm); W: Sample mass (g)).

(3) Determination of Superoxide Dismutase (SOD) Activity.

SOD activity was detected using the Personalbio G0101W SOD Assay Kit (WST-8 method) with a microplate reader (Grace Biotechnology, Suzhou, China). A 0.1 g maize leaf sample was weighed, 1 mL of extraction buffer was added, and the mixture was homogenized in an ice bath at 4 °C. It was centrifuged at 12,000 rpm at 4 °C for 10 min, and the supernatant was collected as the test solution. Prior to measurement, Reagents I, III, and IV from the kit were preheated in a 25 °C water bath for at least 5 min. Reagent IV was thoroughly mixed before use.Reagents were added sequentially according to the system: 280 μL of Reagent I was added to the sample tube, control tube, and blank tube 1. 60 μL of Reagent II was added to the sample tube and blank tube 1, while an equivalent volume of distilled water was added to the control tube and blank tube 2. 60 μL of the test sample was added to both the sample tube and the control tube, whereas no sample was added to the blank tubes. Finally, 30 μL of Reagent III and 320 μL of Reagent IV were added to all tubes. After mixing, the tubes were left to stand in the dark at a room temperature of 25 °C for 30 min. The absorbance of each tube was measured at 450 nm using a microplate reader to calculate SOD activity.

Inhibition Rate (%)=[(A blank 1−A blank 2)−(A sample−A control)]/(A blank 1−A blank 2)×100%.

Calculated based on sample fresh weight: SOD Activity (U/g fresh weight)}=[Inhibition Rate÷(1−Inhibition Rate)×V2]÷(W×V1÷V)×D=12.5×Inhibition Rate÷(1−Inhibition Rate)÷W×D.

(V: Volume of extraction buffer added (1 mL); V1: Sample volume added to the reaction system (0.06 mL); V2: Total volume of the reaction system (0.75 mL); D: Sample dilution factor (1 if undiluted); W: Sample mass (g)).

(4) Determination of Peroxidase (POD) Activity.

POD activity was determined using the Personalbio G0107F Peroxidase Assay Kit (visible colorimetric method) (Grace Biotechnology, Suzhou, China). A 0.1 g maize leaf sample was weighed, 1 mL of extraction buffer was added, and the mixture was homogenized in an ice bath. After centrifugation at 12,000 rpm at 4 °C for 10 min, the supernatant was kept in an ice bath for testing. Reagents I, II, and III were equilibrated to 25 °C in advance.To a cuvette, 40 μL of the test sample, 160 μL of Reagent I, 560 μL of Reagent II, and 40 μL of Reagent III were added sequentially. After rapid mixing, the initial absorbance (A1) was immediately measured at 470 nm using a spectrophotometer. Exactly 1 min later, the second absorbance (A2) was recorded, and ΔA was calculated as A2–A1.

(1) POD Activity (U/g fresh weight)=ΔA÷(W×V1÷V)÷0.5÷T=50×ΔA÷W.

(V: Volume of extraction buffer added (1 mL); V1: Sample volume added (0.04 mL); T: Reaction time (1 min); W: Sample mass (g)).

(5) Determination of Catalase (CAT) Activity.

CAT activity was determined using the Personalbio G0105F Catalase Assay Kit (visible colorimetric method) (Grace Biotechnology, Suzhou, China). A 0.1 g maize leaf sample was weighed, mixed with 1 mL of extraction buffer, and homogenized in an ice bath. It was centrifuged at 12,000 rpm at 4 °C for 10 min, and the supernatant was kept on ice for testing. The spectrophotometer was set to 510 nm and zeroed with distilled water.Blank tube preparation: 80 μL of Reagent I, 20 μL of Reagent II, and 100 μL of Reagent III were mixed. 10 μL of this mixture was taken for the colorimetric reaction, and the absorbance was recorded as A blank.Sample reaction: 10 μL of the test sample, 70 μL of Reagent I, and 20 μL of Reagent II were added to an EP tube. After mixing, the reaction proceeded precisely for 5 min at 25 °C. Then, 100 μL of Reagent III was added and mixed. 10 μL of this mixture (if the solution was turbid, it was centrifuged at 8000 rpm for 10 min to obtain the supernatant) was taken for the colorimetric reaction.Colorimetric system: 10 μL of the aforementioned mixture was combined with 900 μL of Reagent I and 290 μL of Reagent IV. After mixing and reacting at 25 °C for 5 min, the solution was transferred to a cuvette. The absorbance was measured at 510 nm using a spectrophotometer, and ΔA was calculated as A blank-A test. Based on the standard curve $y=0.2091x-0.0026(R^{2}=0.9999)$, the CAT activity was calculated using the following formula:

CAT Activity (U/g fresh weight)=[(ΔA+0.0025)÷0.2093]÷(W×V1÷V)÷T×D=95.6×(ΔA+0.0025)÷W×D.

(V: Volume of extraction buffer added (1 mL); V1: Sample volume added (0.01 mL); T: Reaction time (5 min); W: Sample mass (g); D: Dilution factor).

#### 2.5.3. Determination of Rhizosphere Soil Microorganisms

Samples of maize rhizosphere microorganisms were collected 1 day prior to the drought treatment (30 June, late whorl stage), 60 days after treatment, and 75 days after treatment (maturity), labeled sequentially as A, B, and C. The sample numbers for each treatment were designated as JN01A, JN01B, JN01C, JD01A, JD01B, JD01C, JN03A, JN03B, JN03C, JD03A, JD03B, and JD03C.

Rhizosphere soil samples were collected using the root-shaking method. Specifically, the entire maize root system was carefully uprooted along with the surrounding soil. After gently shaking off the loosely attached bulk soil, the soil tightly adhering to the root surface was brushed off using a sterile brush to serve as the rhizosphere sample. Each treatment was performed in biological triplicate. The collected samples were placed into sterile 15 mL centrifuge tubes, transported in a styrofoam cooler containing dry ice, and subsequently stored at −80 °C until further analysis.

Rhizosphere Soil DNA Extraction and Metagenomic Sequencing: Total microbial genomic DNA was extracted from the maize rhizosphere soil using the MagBeads FastDNA Kit for Soil (Cat. No. 116564384; MP Biomedicals, San Diego, CA, USA) in strict accordance with the manufacturer’s protocol. DNA concentration was quantified using a Qubit™ 4 Fluorometer with the 1 × dsDNA HS Assay Kit (Invitrogen, San Diego, CA, USA), while DNA integrity was verified via 1% agarose gel electrophoresis. Qualified DNA samples were subsequently used to construct metagenomic shotgun libraries with an insert size of 400 bp using the Illumina TruSeq Nano DNA LT Library Preparation Kit. Finally, paired-end sequencing (PE150) was performed on the Illumina NovaSeq platform (Illumina, San Diego, CA, USA).

Data Quality Control, Assembly, and Gene Prediction: Raw sequencing reads were processed using fastp (v0.23.2) to remove adapter sequences. A sliding window approach (5 bp window size) was employed to trim low-quality reads with an average quality score below Q 20, sequences shorter than 50 bp, or those containing ambiguous bases. To eliminate host contamination from the rhizosphere samples, the resulting clean reads were aligned against the maize reference genome using Minimap2 (v2.24-r1122). The filtered reads then underwent de novo assembly using MEGAHIT (v1.1.2), retaining contigs ≥ 300 bp. Open reading frames (ORFs) and coding regions within the assembled contigs were predicted using Prodigal (v2.6.3). The predicted protein sequences were subsequently dereplicated using the easy-linclust module in MMseqs2 (v 13.45111) at thresholds of 95% sequence identity and 90% coverage to generate a non-redundant gene/protein catalog.

Taxonomic and Functional Annotation: For taxonomic classification, the clean reads were directly mapped against the NCBI non-redundant (nt) database via high-precision k-mer matching using Kraken 2 (v2.0.8-beta). Bracken was then applied to re-estimate taxonomic abundances at various levels, including genus and species, based on a Bayesian algorithm. For functional annotation, the non-redundant protein catalog was aligned to the KEGG database using the search module of MMseqs2 to generate functional abundance profiles. Finally, read mapping and quantification were performed using Strobealign (v0.12.0) and featureCounts (v2.0.3). The normalized relative abundances of both taxonomic and functional features were expressed as Transcripts Per Kilobase Million (TPM) for downstream statistical analyses.

#### 2.5.4. Determination of Soil Water Content

Volumetric soil water content (%) was measured on the fourth day after irrigation, between 9:00 and 11:00 AM, using a TDR150 soil moisture meter (Spectrum Technologies, Auror, IL, USA). For each treatment, measurements were recorded at depths of 7.5 cm and 20 cm using the corresponding probes. Three measurement points were randomly selected per treatment, situated 20 cm away from the maize plants. The values from these three points were averaged to determine the final soil water content for each treatment.

### 2.6. Data Analysis

Experimental data were processed and statistical functions calculated using Microsoft Excel. ANOVA and Tukey’s HSD (Honestly Significant Difference) post hoc tests were performed using SPSS 27 software to determine the significance of differences. Correlation analysis and graphing were carried out using the Spearman method in Origin 2021.

Results regarding soil microbial diversity were obtained from the Personal Biotechnology GenesCloud Platform accessed on 21 October 2025 (https://www.genescloud.cn). The GenesCloud visualization software was utilized to analyze sample composition, function, and physiological-biochemical correlations.

## 3. Results

### 3.1. Effects of Drought Treatment During the Twelfth Leaf Stage on Maize Yield and Related Major Agronomic Traits

#### 3.1.1. Effects of Drought Stress During the Ear Stage on Maize Yield and Its Components

Drought stress impacted maize yield and its yield components, although the effects varied depending on the variety (Table 3). The JN01 treatment exhibited the highest EY (17.40 t/hm^2^), GW, KPS and HGW. These values were significantly higher than those of the JN03 and JD03 treatments (between which there was no significant difference). Furthermore, the parameters for the JD01 treatment were significantly lower than those of all the aforementioned treatments. This indicates that drought stress had a significant effect on the yield and its components for Variety 01 (*p* < 0.05), demonstrating its weak drought tolerance. Conversely, the impact on Variety 03 was relatively minor; apart from significant differences in HGW and KPS, the remaining indicators did not reach statistical significance, suggesting that Variety 03 possesses stronger drought tolerance.

#### 3.1.2. Effects of Drought Stress During the Ear Stage on Ear Traits of Maize

Drought also affects the ear traits of maize, but the effects vary depending on the variety.

As shown in Table 4, drought stress had a significant effect on EL, BTL, ED, NGR, and TR. Among these, JN01 exhibited the greatest EL, BTL, ED, NGR, and TR, all of which were significantly higher than those of the other treatments. With the exception of EL, there were no significant differences between JN03 and JD03 for the other four indicators. This indicates that drought stress had a significant effect on the ear traits of variety 01, whereas the effect on variety 03 was not significant. Under drought treatment, no significant differences were observed, whilst the remaining indicators reached significant or highly significant levels.

#### 3.1.3. Analysis of Variance and Correlation Analysis of Yield and Related Traits During the Ear Stage

**Figure 1:**
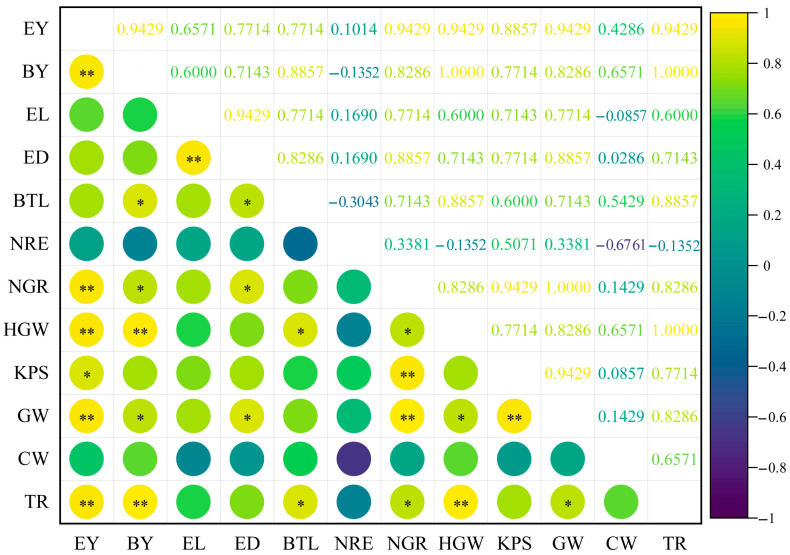
Results of correlation analysis for ten agronomic traits of maize. Note: The colour of the dots represents the sign and magnitude of the correlation coefficient; yellow indicates a positive correlation, purple indicates a negative correlation, and the darker the colour, the stronger the correlation. * indicates *p* ≤ 0.05, ** indicates *p* ≤ 0.01.

A correlation analysis was conducted on 10 agronomic traits, including yield, for the maize varieties. As shown in Figure 1, the EY of maize was found to have a highly significant positive correlation (r = 0.9429) with BY, NGR, HGW, GW, and TR, and a significant correlation (r = 0.8857) with KPS. BY showed a highly significant positive correlation with HGW and TR. It also showed a significant positive correlation with BTL, GW, and NGR.

### 3.2. Effects of Drought Treatment on Physiological and Biochemical Traits

Under drought stress, the activity of the antioxidant enzyme system (SOD, POD, CAT) decreased 60 days after treatment compared to before treatment, whilst MDA accumulation increased, indicating exacerbated oxidative damage (Table 5). The drought-tolerant variety (03) exhibited a smaller decline in enzyme activity and lower MDA accumulation, whilst demonstrating a stronger capacity for Pro accumulation, thereby exhibiting superior drought tolerance compared to the non-drought-tolerant variety (01).

### 3.3. Analysis of Soil Rhizosphere Microbial Community Composition and Diversity Under Drought Treatment

#### 3.3.1. Analysis of Rhizosphere Microbial Community Composition at the Phylum Level

**Figure 2:**
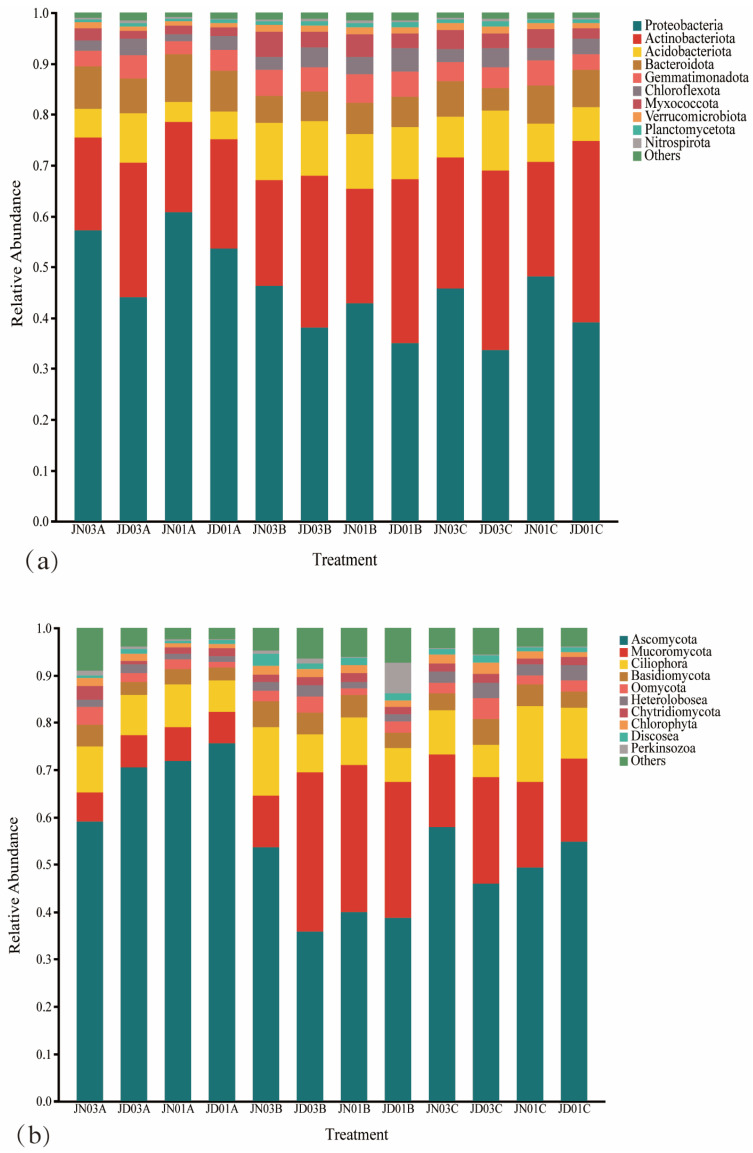
Distribution of relative abundance of microbial phyla in the rhizosphere soil of maize under different treatments. Note: (**a**) Relative abundance of bacterial communities at the phylum level. (**b**) Relative abundance of fungal communities at the phylum level. JN and JD represent two treatment methods, respectively; cultivar numbers 01 and 03 denote drought-sensitive and drought-tolerant maize varieties, respectively. The vertical axis indicates relative abundance (%), and the horizontal axis shows the treatment group labels. Different colours in the legend represent different microbial phyla, and “Others” represents the sum of phyla with relatively low abundance.

Drought stress affects the diversity and composition of the rhizosphere microbial community in crops [24]. Figure 2 shows the distribution of bacteria (2a) and fungi (2b) at the phylum level in the maize rhizosphere.

Regarding the phylum-level composition of the bacterial community, the dominant phyla were largely consistent across treatment groups, although differences in relative abundance were observed (Figure 2a). The top ten bacterial phyla in each sample, listed from highest to lowest abundance, were: Proteobacteria, Actinobacteriota, Acidobacteriota, Bacteroidota, Chloroflexota, Myxococcota, Gemmatimonadota, Verrucomicrobiota, Planctomycetota, and Nitrospirota. Under drought stress, the abundance of the Proteobacteria phylum decreased in the rhizosphere of both varieties, whilst that of the Actinobacteriota phylum increased; this change intensified with prolonged drought, and the community structure of the drought-sensitive variety 01 underwent more drastic changes.

Regarding the phylum-level composition of the fungal community (Figure 2b), the relative abundances of dominant fungal phyla across the treatment groups, from highest to lowest, were: Ascomycota, Basidiomycota, Mucoromycota, Chytridiomycota, Chlorophyta, Oomycota, Heterolobosea, Ciliophora, Discosea, and Perkinsozoa. As the stress period progressed, the abundance of the Ascomycota phylum decreased in the non-drought-tolerant variety 01, whilst the abundance of the Mucoromycota phylum increased; in contrast, the decline in Ascomycota abundance was less pronounced in the drought-tolerant variety than in the non-drought-tolerant variety.

#### 3.3.2. Analysis of Rhizosphere Microbial Community Composition at the Genus Level

**Figure 3:**
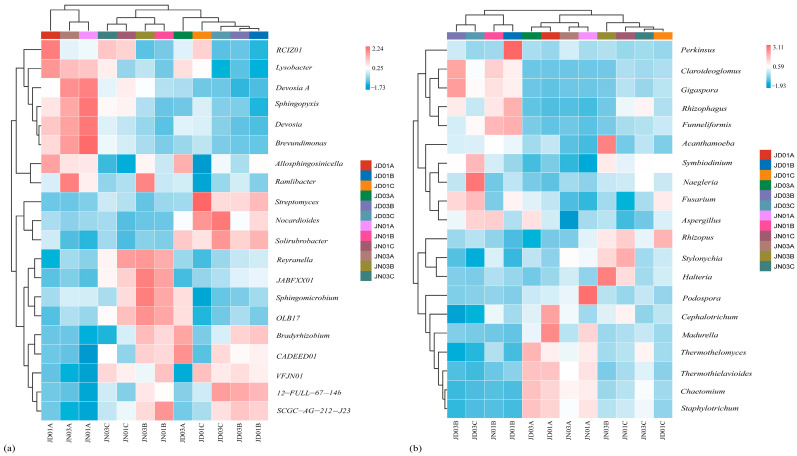
Heatmap showing the distribution of soil microorganism genera in the maize rhizosphere under different treatments. Note: The colour scale represents Z-score normalised relative abundance, with red indicating higher and blue indicating lower relative abundance relative to the mean. (**a**) Top 20 bacterial genera; (**b**) Top 20 fungal genera. Each column represents one biological replicate.

Figure 3 shows heatmaps of the distribution of bacteria (3a) and fungi (3b) at the genus level in the maize rhizosphere.

The clustering results of the bacterial community at the genus level (Figure 3a) show that the drought treatment (JD) and normal treatment (JN) are generally separated. Under drought conditions, the relative abundances of *Lysobacter*, *Streptomyces*, and *Brevundimonas* increased, whilst those of *Bradyrhizobium* and *Sphingomicrobium* decreased. The drought-tolerant variety (03) retained higher abundances of *Nocardioides* and *Sphingopyxis* under drought treatment, exhibiting a more stable rhizosphere bacterial community. These differences became more pronounced as the drought period progressed.

The results of the genus-level fungal community (Figure 3b) clustering heatmap indicate that the drought treatment altered the composition of the rhizosphere fungal community, with arbuscular mycorrhizal fungi (*AMF*)-associated genera, including the endosphere *Rhizophagus* and *Funneliformis*, maintaining higher abundances in the drought-tolerant variety (03), whilst saprophytic or potentially pathogenic fungi such as *Fusarium* and *Aspergillus* increased relatively under drought conditions. As the sampling period extended, the abundance of the non-drought-tolerant variety 01 continued to decline, whereas that of the drought-tolerant variety 03 remained relatively stable.

#### 3.3.3. Effects of Drought Treatment During the Maize Ear Stage on Soil Rhizosphere Microbial Community Diversity

1 Analysis of Alpha Diversity Composition.

**Figure 4:**
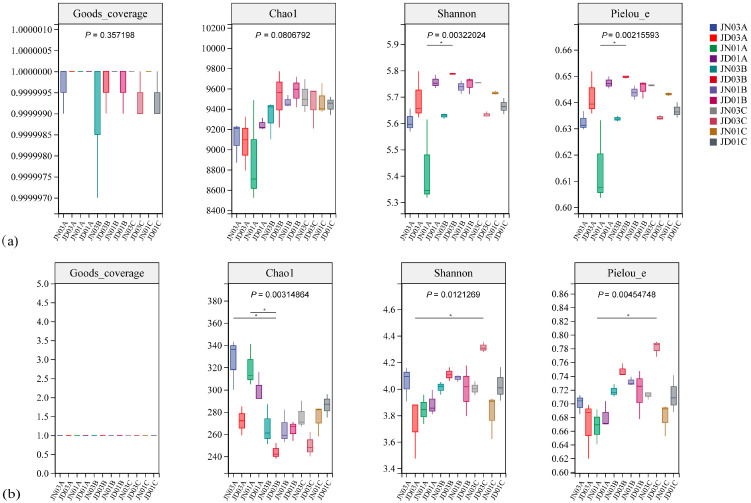
Box plots of alpha diversity indices of soil microorganisms in the rhizosphere of maize. Note: (**a**) Analysis of alpha diversity at the genus level of soil bacteria in the rhizosphere of maize. (**b**) Analysis of alpha diversity at the genus level of soil fungi in the rhizosphere of maize. The horizontal lines in the box plots represent the minimum value, the lower quartile (Q1), the median, the upper quartile (Q3) and the maximum value, in sequence from bottom to top. The *p*-values were obtained from the Kruskal–Wallis test, and * indicates significant differences between groups (*p* < 0.05).

Alpha diversity refers to the species diversity within a single sample (or a specific habitat or community) and is an important indicator for assessing the health, stability, and functional potential of the rhizosphere micro-ecosystem [25] (Figure 4).The Goods_coverage values for all bacterial and fungal samples were close to or equal to 1, and there were no significant differences between groups, indicating that the sequencing depth in this study was sufficient and the results were highly reliable.The Chao1 index for bacteria showed a not significant difference between groups (*p* = 0.0806792), whilst that for fungi was highly significant (*p* = 0.00314864), suggesting that drought stress had a more pronounced effect on fungal species richness. Both bacterial and fungal values were lower in the drought-treated group (JD) than in the normal moisture group (JN), and this difference gradually increased with sampling time. Inter-variety comparisons showed that the decline in bacterial and fungal Chao1 indices was greater in the non-drought-tolerant variety 01 than in the drought-tolerant variety 03 under drought treatment.The differences in the Shannon indices for both bacteria and fungi were significant (*p* = 0.00322024) and (*p* = 0.0121269), respectively, both indicating that the drought treatment significantly reduced the species diversity of rhizosphere bacterial and fungal communities. As the duration of stress increased, the indices in the drought-treated groups, particularly the non-drought-tolerant variety JD01, declined markedly in the later stages compared to the drought-tolerant varieties, whilst the values in the normal treatment group remained relatively stable throughout all periods.The differences in the Pielou_e indices for bacteria and fungi were both highly significant (*p* = 0.00215593) and (*p* = 0.00454748), respectively, and both showed significant markers. The Pielou_e values in the drought-treated group were all lower than those in the normal treatment group. Furthermore, the evenness of both bacteria and fungi reached their lowest levels in the later stages of the drought treatment (Phase C). The drought-tolerant variety (03) exhibited higher bacterial and fungal levels than the non-drought-tolerant variety (01) at all stages.

2 Analysis of Beta Diversity Composition.

**Figure 5:**
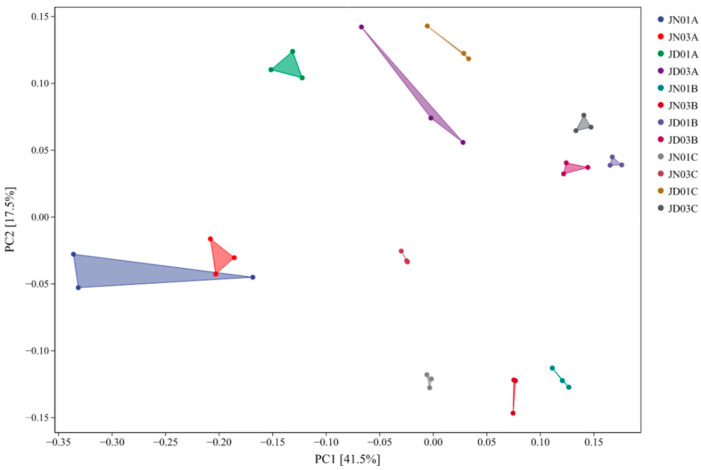
Ordination diagram of beta diversity of soil microorganisms in the rhizosphere of maize.

Beta diversity, or “diversity between habitats,” is used to measure the degree of difference in microbial community composition between different samples or ecosystems [26]. Beta-diversity analysis was performed on the rhizosphere microbial communities of maize across different treatments at the genus level; the results are shown in Figure 5.

As shown in Figure 5, the PC1 and PC2 axes explained 41.5% and 17.5% of the total variation in the rhizosphere soil microbial community, respectively; samples from the normal moisture and drought stress treatments were clearly separated along the PC1 axis. Under drought conditions, as the duration of stress increased, the community structure formed a distinct temporal gradient along the PC2 axis. Throughout the drought cycle, the rhizosphere microbial community of the drought-tolerant maize variety (03) remained highly stable, with concentrated sample point distribution and a closer proximity to the normal moisture group; conversely, the distance between the non-drought-tolerant variety (01) and the normal moisture group increased as the duration of drought prolonged.

### 3.4. Functional Analysis of the Soil Rhizosphere Microbial Community

#### 3.4.1. Results of LEfSe Analysis

**Figure 6:**
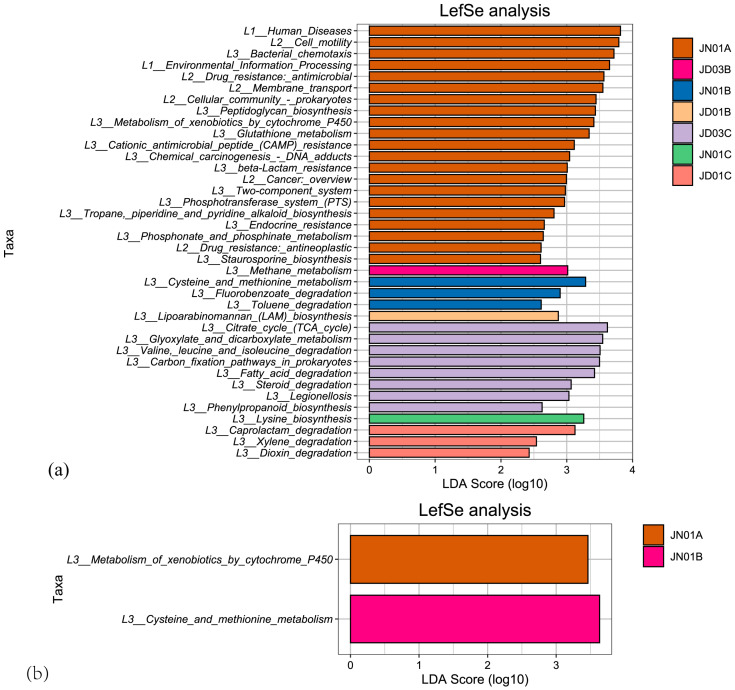
LEfSe analysis of soil rhizosphere microorganisms in maize.Note: LEfSe analysis identifying significant biomarkers across different groups. (**a**) Bacterial community; (**b**) Fungal community. The histogram displays the features (e.g., functional pathways) with significant differential abundance between groups. The length of the bar represents the log10-transformed LDA (Linear discriminant analysis) score, indicating the effect size of each feature. The colors of the bars correspond to the specific groups where these features are significantly enriched.

The results of the bacterial LEfSe analysis (Figure 6a) show that the normal treatment of JN01A exhibited the highest number of enriched differential functional pathways, covering multiple pathways related to human diseases, cell motility, bacterial chemotaxis, environmental information processing, drug resistance (antimicrobial and antitumour), membrane transport, peptidoglycan biosynthesis, glutathione metabolism, and phosphotransferase systems, with generally high LDA scores. Upon entering Phase B, the differential effects of treatment and strain gradually diverged. In the control treatment of non-drought-tolerant strains, pathways such as methane metabolism, cysteine and methionine metabolism, fluorobenzoic acid degradation and toluene degradation were enriched, whilst the drought-treated group showed enrichment in pathways such as the citrate cycle, glyoxylate and dicarboxylic acid metabolism, branched-chain amino acid degradation, prokaryotic carbon fixation, fatty acid degradation and steroid degradation. In drought-treated samples of drought-tolerant varieties, enrichment was observed only in the staurosporine biosynthesis pathway. By stage C, drought-treated drought-tolerant varieties were enriched in the phenylpropanoid biosynthesis and lysine biosynthesis pathways, whilst non-drought-tolerant varieties showed a trend of enrichment in the xylene degradation and dioxin degradation pathways; under normal conditions, non-drought-tolerant varieties exhibited specific enrichment in the caprolactam degradation pathway.

The results of the fungal LEfSe analysis (Figure 6b) show that, across all treatments, only JN01A and JN01B exhibited differentially expressed functional pathways. JN01A was enriched in the metabolism of xenobiotics by cytochrome P450 pathway, whilst JN01B was enriched in the cysteine and methionine metabolism pathways.

#### 3.4.2. Analysis of Species Contribution to the Community

**Figure 7:**
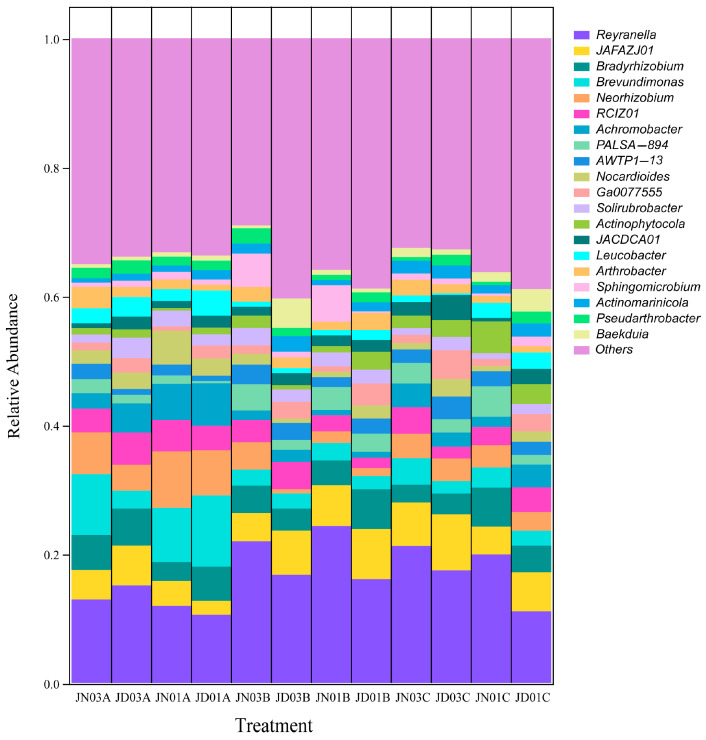
Contribution degree of microbial species in the maize rhizosphere.

The results of the community species contribution analysis (Figure 7) indicate that the genus *Reyranella* was the most significant contributing group to the rhizosphere bacterial community across all treatment and variety combinations. Species contributions were similar across treatments in the early stages, but differences between groups widened in the later stages, with the genus *Pseudarthrobacter* or *Achromobacter* enriching in drought-tolerant varieties under drought treatment. By harvest (C), the abundance of *Reyranella* increased further, whilst the contribution of groups such as *Bradyrhizobium* and *Sphingomicrobium* was higher in the drought treatment than in the normal treatment; moreover, the drought-tolerant variety 03 contributed more groups than the less drought-tolerant variety 01.

#### 3.4.3. Co-Occurrence Network Analysis

**Figure 8:**
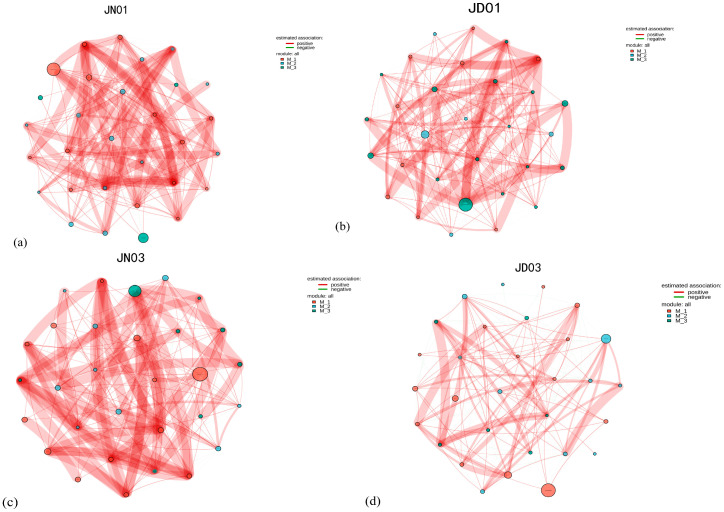
Co-occurrence network structure of bacterial communities in the maize rhizosphere. Note: Nodes represent bacterial taxa, size indicates connectivity; red/green lines represent positive/negative correlations, and the thickness of the lines indicates the strength of the correlation. (**a**) JN01, (**b**) JD01, (**c**) JN03, (**d**) JD03.

The bacterial co-occurrence network analysis is shown in Figure 8. In all four networks, red connections (positive correlations) predominate, whilst negative correlations (blue/dark connections) are extremely rare. The network density in the normal treatment was significantly higher than that in the drought treatment; connections between nodes were tighter, network complexity was higher, and the number of key nodes and the integrity of the network framework were greater in the drought-tolerant variety than in the non-drought-tolerant variety.

**Figure 9:**
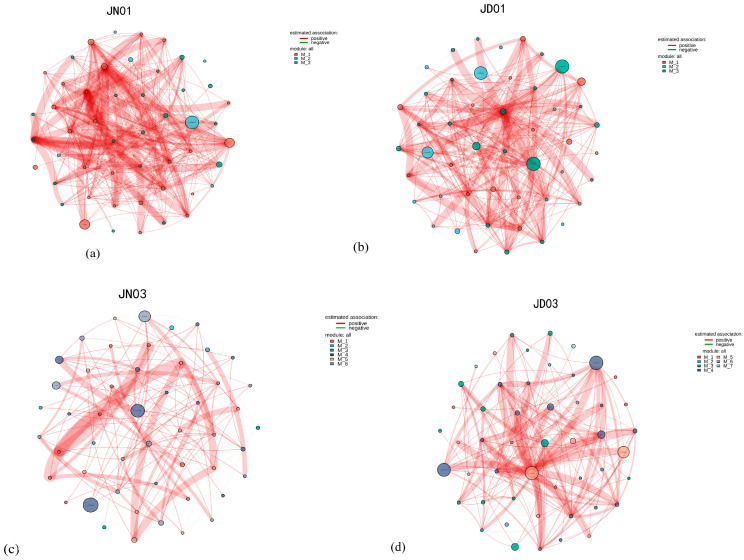
Co-occurrence network structure of the fungal community in the maize rhizosphere. Note: Nodes represent fungal taxa, and the size indicates the connectivity; red/green lines represent positive/negative correlations, and the thickness of the lines indicates the strength of the correlation. (**a**) JN01, (**b**) JD01, (**c**) JN03, (**d**) JD03.

The four networks analysed in the fungal co-occurrence network analysis (Figure 9) are similarly dominated by red lines (positive correlations), but compared to the bacterial networks, there are more blue nodes (different taxonomic groups) and the differences in node size are more pronounced.The number of nodes and link density in the drought-treated networks were generally higher than in the control treatment, contrary to the trend observed in the bacterial networks. The JD03 network had more nodes and links than JD01, and the large blue nodes were more prominent.

### 3.5. Correlation Analysis of Soil Rhizosphere Microorganisms and Physiological and Biochemical Parameters

#### 3.5.1. Correlation Between Bacterial Agronomic Traits and Enzyme Activity Indices

**Figure 10:**
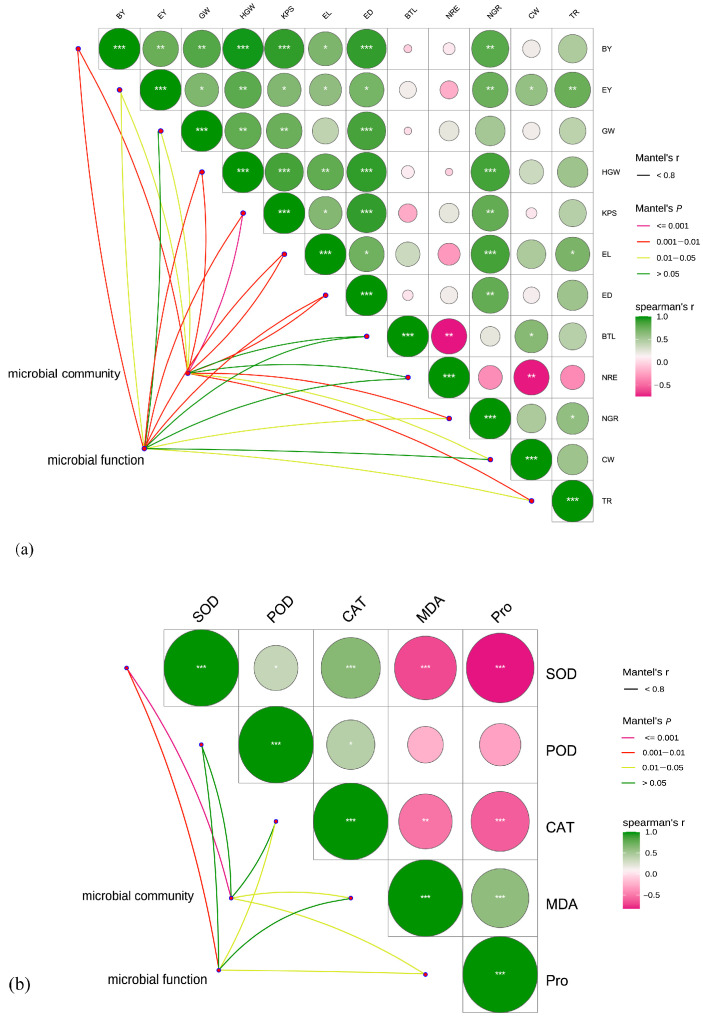
Mantel test assessing relationships between the maize bacterial rhizosphere community/function and agronomic/physiological indicators. Note: (**a**) Association between bacterial community/function and agronomic traits; (**b**) Association between bacterial community/function and physiological and biochemical indicators. The colour and size of the circles represent the Spearman correlation coefficient, and the colour of the lines indicates the significance level of the Mantel test. Asterisks within the circles denote the statistical significance levels of Spearman’s correlation: * *p* < 0.05, ** *p* < 0.01, and *** *p* < 0.001.

Spearman’s correlation analysis showed that bacteria were significantly positively correlated with most agronomic trait indices (Figure 10a). The Mantel test indicated that the association between bacterial community composition and KPS was extremely significant (*p* ≤ 0.001), whilst associations with BY, HGW, KPS, EL, ED, NGR, and TR were extremely significant (0.001 < *p* < 0.01), and significant associations were observed with EY, GW, and CW (0.01 < *p* < 0.05). Microbial function showed a highly significant association with BY, HGW, KPS, EL, and ED (0.001 < *p* < 0.01), and a significant association with EY, NGR, and TR (0.01 < *p* < 0.05); associations with the remaining indicators were not significant.

Among physiological and biochemical indicators (Figure 10b), the Mantel test indicated that bacterial community composition was extremely significantly associated with SOD (*p* ≤ 0.001) and significantly associated with CAT and MDA (0.01 < *p* < 0.05). Bacterial function was extremely significantly associated with SOD (*p* ≤ 0.001) and significantly associated with CAT and Pro (0.01 < *p* < 0.05).

**Figure 11:**
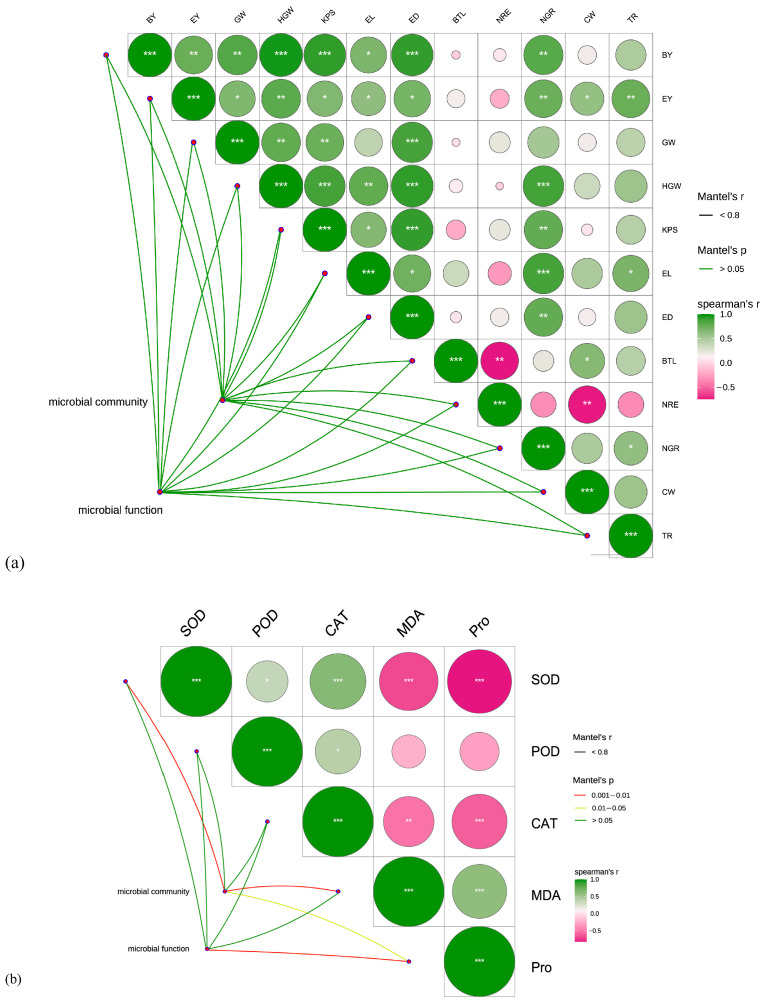
Mantel test assessing relationships between the maize fungal rhizosphere community/function and agronomic/physiological indicators. Note: (**a**) Association between fungal community/function and agronomic traits; (**b**) Association between fungal community/function and physiological and biochemical indicators. The colour and size of the circle represent the Spearman correlation coefficient, and the colour of the line represents the significance level of the Mantel test. Asterisks within the circles denote the statistical significance levels of Spearman’s correlation: * *p* < 0.05, ** *p* < 0.01, and *** *p* < 0.001.

In the analysis of associations between fungal microbial communities and agronomic traits (Figure 11a), Spearman’s correlation analysis revealed significant positive correlations among most agronomic trait indicators. The results of the Mantel test indicated that, under drought stress, fungal community structure and function had no significant effect on EY or ear-related traits.

In the analysis of the association between antioxidant enzyme activity and fungal community and function (Figure 11b), the three antioxidant enzymes SOD, POD, and CAT showed highly significant positive correlations with each other. MDA was negatively correlated with SOD, POD, and CAT, whilst proline was positively correlated with the antioxidant enzyme system and negatively correlated with MDA. The results of the Mantel test indicate that the association between fungal community composition and SOD and MDA reached a highly significant level (0.001 < *p* < 0.01), and a significant association was observed with Pro (0.01 < *p* < 0.05). Fungal function was associated with Pro at a highly significant level (0.001 < *p* < 0.01).

#### 3.5.2. RDA Analysis

**Figure 12:**
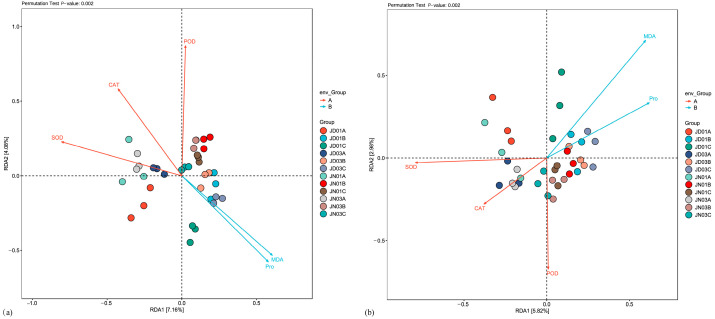
RDA of microorganisms in the rhizosphere soil of maize. Note: (**a**) Bacterial community; (**b**) Fungal community. The horizontal and vertical dashed lines in both panels represent the zero reference axes intersecting at the origin.The horizontal and vertical dashed lines represent the zero reference axes (x = 0 and y = 0) intersecting at the origin for this ordination space.

The results of the RDA permutation test for bacterial and fungal communities showed *p*-values of 0.002 in both cases, indicating that environmental factors have a significant explanatory power regarding the structure of rhizosphere microbial communities. In the bacterial RDA ordination plot (Figure 12a), the effects of SOD, CAT, and POD activities (Period A) and MDA and Pro levels (predominantly Periods B and C) on community structure showed clear differentiation: they were negatively correlated with bacterial communities, whereas MDA and Pro levels were positively correlated with bacterial communities.

In the fungal RDA ordination plot (Figure 12b), MDA and Pro content had a significant effect on the fungal community and showed a strong positive correlation with community structure, whilst the effects of SOD, CAT, and POD activities were weaker; among these, POD activity showed a negative correlation with the fungal community, and samples from different treatments were more clearly separated along the gradient of stress response indicators.

## 4. Discussion

### 4.1. Effects of Drought on Maize Yield and Major Agronomic Traits

Extensive global-scale studies confirm that maize yield decreases significantly under drought stress [27]. Water deficit at any growth stage of maize disrupts critical physio-biochemical processes, which leads to stunted plant growth and source-sink imbalance, ultimately resulting in a substantial reduction in yield [28]. Drought during the ear stage of maize significantly increases barren tip length, reduces the number of kernels per ear and 100-kernel weight, leading to a decrease in yield. Drought stress also significantly reduces the 100-kernel weight, ear weight, shelling percentage, and ear length of maize [2]. The results of this study indicate that drought stress at the late whorl stage significantly reduced the biological and economic yields of the drought-sensitive variety Zhongdan 808, as well as ear length, ear diameter, kernel number per row, 100-kernel weight, and cob diameter, while significantly increasing barren tip length. This is consistent with the findings of Cairns et al. (2012), found that drought-induced reductions in yield and its components are less pronounced in drought-tolerant genotypes compared to susceptible ones [29]. These results not only confirm that Zhongdan 808 is drought-sensitive [22] and NK718 is drought-tolerant [21], but also further demonstrate that late-stage drought during ear development has a minimal impact on the growth, development, and yield of drought-tolerant varieties.

From a physiological and biochemical perspective, drought stress typically triggers an imbalance in reactive oxygen species (ROS) and lipid peroxidation damage to cell membranes within plants. In this study, drought treatment led to a decrease in the activity of antioxidant enzymes such as SOD, POD, and CAT in both varieties compared to pre-treatment levels, alongside a massive accumulation of MDA. This suggests that prolonged, severe drought stress may have exceeded the plants’ intrinsic ROS scavenging thresholds, resulting in damage to the cell membrane system. Concurrently, plants activate osmotic adjustment mechanisms to counteract dehydration stress [30]. Previous studies have demonstrated that drought-tolerant varieties can maintain elevated proline content and higher antioxidant enzyme activity, thereby modulating reactive oxygen species homeostasis under stress [31]. The results of this study are consistent with these findings: the drought-tolerant variety NK718 exhibited a smaller decline in enzyme activity, significantly lower MDA accumulation than Zhongdan 808, and a stronger ability to accumulate proline (Pro). This indicates that NK718 possesses a higher physiological damage threshold and stronger osmotic adjustment adaptability.

### 4.2. Effects of Drought on Soil Microorganisms in the Maize Rhizosphere

Against the backdrop of the increasingly urgent need for sustainable agricultural development, research into plant rhizosphere soil microorganisms has become a focal point in agroecology [32].

In response to water deficit, plants actively drive the structural reorganization of rhizosphere microbial communities [33]. This study found that drought stress significantly reduced the richness (Chao1) and diversity (Shannon index) of the rhizosphere soil microbial community. This aligns with the findings of Kristy et al. (2022), who revealed that drought-tolerant genotypes adapt to chronic drought stress by selectively enriching for growth-promoting microorganisms in their rhizosphere microbiome [34]. At the phylum level, Proteobacteria and Actinobacteriota were confirmed as core taxa in the maize rhizosphere [35]. As drought stress intensified, we observed a significant decline in the relative abundance of Proteobacteria, while the abundance of Actinobacteriota increased significantly. This succession pattern is highly consistent with the conclusions of Chodak et al. (2015) [36]. As reviewed by Fan et al. (2023), drought stress reshapes the structure of the rhizosphere microbial community, and plants can enhance their drought resistance by selectively recruiting specific root-associated bacteria [37]. Gram-positive bacteria, such as Actinobacteriota, typically possess thicker peptidoglycan cell walls, enabling them to withstand dehydration stress more effectively; whereas Gram-negative bacteria, such as the copiotrophic Proteobacteria, are more sensitive to drastic changes in the moisture environment. Furthermore, the drought-tolerant variety NK718 maintained higher abundances of *Nocardioides*, *Sphingopyxis*, and *arbuscular mycorrhizal fungi* (*AMF*)-associated genera under drought conditions. *AMFs* have been widely demonstrated to improve plant water and nutrient uptake via their hyphal networks and mitigate drought-induced oxidative stress, suggesting that drought-tolerant varieties may assist in drought resistance by selectively recruiting specific beneficial microorganisms to maintain cell membrane integrity and photosynthetic efficiency [38].

In terms of functional analysis, bacterial LEfSe analysis revealed that the interaction between drought treatment and variety drought tolerance exerted an increasingly pronounced influence on the functional structure of the rhizosphere bacterial community as the growth stage progressed. Under drought conditions, drought-tolerant varieties enhanced their adaptability by enriching metabolic pathways closely associated with plant stress resistance, such as phenylpropanoid biosynthesis and lysine biosynthesis. Species contribution analysis indicated that drought-tolerant varieties exhibited a stronger ability to synergistically recruit multifunctional bacterial taxa under drought stress, which may be one of the key mechanisms for maintaining the stability and functional diversity of the rhizosphere microbiome. Studies have found that rhizosphere microbial community function is closely related to crop drought tolerance, and plants can enhance their adaptation to drought stress by regulating rhizosphere microbial functions. Analysis of microbial co-occurrence networks provides a visual representation of symbiotic interactions among species within a community. This study demonstrates that drought stress leads to a loosening of bacterial co-occurrence networks, with a reduction in the number of connections. The drought-tolerant variety NK718 was able to maintain a more stable rhizosphere bacterial co-occurrence network structure under drought stress, while the degree of reorganization and strengthening of its fungal network was more pronounced. However, fungal networks exhibited higher stability and resistance than bacterial networks under drought stress, which reflects an ecological adaptation strategy whereby fungi utilize their extensive hyphal networks to bridge air-filled soil pores in order to obtain water and nutrients [39].

Plant physiological responses and the rhizosphere microecology are not isolated events. In this study, the results of Spearman correlation analysis and the Mantel test indicated that microbial community composition was significantly associated with yield-related agronomic traits and antioxidant enzyme activity indices. RDA further validated that environmental factors have significant explanatory power for rhizosphere fungal community structure, and that MDA and POD are key enzymatic indicators influencing fungal community composition; moreover, the impact of drought stress on the rhizosphere fungal community structure is significantly stronger than on the bacterial community. Under drought stress, crop root exudates, phytohormones, and rhizosphere microorganisms form a complex interactive network to collectively combat drought and help host plants adapt to the stress [40]. In this study, differences in the response patterns of varieties with varying drought tolerance became increasingly pronounced as the duration of stress extended, indicating that a variety’s drought tolerance is closely related to the structural characteristics and functional composition of its rhizosphere microbial community.

In summary, there are significant differences among maize varieties with varying levels of drought tolerance in terms of yield, physiological and biochemical responses, and rhizosphere microbial community dynamics under drought stress. Drought-tolerant varieties not only maintain higher antioxidant enzyme activity and osmotic adjustment capacity at the physiological level but also enhance their adaptability to drought stress by regulating the structure and function of the rhizosphere microbial community. Although this study has revealed a strong correlation between plant phenotypes and microecological responses, the precise causal mechanisms by which the microbiome mediates drought resistance remain to be confirmed in future studies through specific microbiome isolation and inoculation experiments. This study provides a new perspective on “plant-microbe interactions” for elucidating the mechanisms of maize drought resistance, and offers a theoretical basis for achieving stable maize yields under drought conditions through targeted microbiome regulation in the future.

## 5. Conclusions

Drought stress during the maize ear stage (late whorl stage) significantly reduced the economic yield of maize. Specifically, it had a significant impact on ear length, ear diameter, kernel number per row, 100-kernel weight, and barren tip length in drought-sensitive varieties (NK718); in drought-tolerant varieties (NK718), the effect was not significant for any of these parameters except ear length. Drought led to a decrease in antioxidant enzyme (SOD, POD, CAT) activities, an increase in malondialdehyde (MDA) accumulation, and elevated proline content. Drought-tolerant varieties exhibited a smaller decline in antioxidant enzyme activities, reduced accumulation of the lipid peroxidation marker MDA, and a stronger capacity for proline accumulation, demonstrating greater antioxidant and osmotic adjustment capabilities.

Drought stress significantly altered the composition, diversity, and function of bacterial and fungal communities in the maize rhizosphere. At the phylum level, drought stress affected the distribution of the dominant taxa, Proteobacteria and Actinobacteriota, in the maize rhizosphere soil. As the duration of drought stress increased, the relative abundances of Proteobacteria and Bacteroidetes decreased significantly, while those of Actinobacteriota increased significantly. At the genus level, the relative abundances of *Lysobacter* and *Streptomyces* increased, while that of *Bradyrhizobium* decreased. Among fungi, the abundance of *Ascomycota* decreased, while that of *Mucoromycota* increased. Drought reduced the Chao1 and Shannon indices of rhizosphere microorganisms, with a more pronounced effect on fungal diversity. Under drought conditions, the drought-tolerant variety 03 (NK718) maintained higher community stability and species richness; Beta diversity analysis indicated that its sampling points clustered closer to those of the well-watered group. Functional analysis indicated that the drought-tolerant variety (NK718) was enriched in beneficial metabolic pathways such as phenylpropanoid biosynthesis. Co-occurrence network analysis revealed that drought reduced the complexity of bacterial networks but enhanced that of fungal networks, and exhibited greater network robustness and reorganizational capacity. Correlation analysis further confirmed that microbial community composition is significantly correlated with agronomic traits such as yield and ear-kernel weight, as well as physiological indicators such as SOD and MDA.

The findings of this study lay a solid foundation for in-depth research into the mechanisms underlying root-soil bacteria interactions and the enhancement of maize drought tolerance and yield. Furthermore, they provide a theoretical basis for selecting drought-tolerant maize varieties to improve soil microbial community structures, ultimately enhancing maize drought tolerance, yield, and grain quality in future applications.

## Figures and Tables

**Table 1:** Soil moisture content results in the experimental site.

Water Content	JN01 (%)		JN03 (%)		JD01 (%)		JD03 (%)	
Deep	7.5 cm	20 cm	7.5 cm	20 cm	7.5 cm	20 cm	7.5 cm	20 cm
24 April	21.96	36.39	22.24	36.44	21.42	36.04	21.47	35.91
15 May	20.52	27.84	20.01	27.34	20.16	27.06	21.80	28.79
5 June	14.39	22.20	14.36	22.00	15.47	23.41	15.17	23.96
19 June	12.05	19.59	12.24	19.99	12.06	14.93	12.35	14.81
3 July	12.66	17.77	12.90	17.99	11.27	13.10	10.37	13.72
7 August	12.72	17.47	12.32	18.24	6.26	8.53	6.47	9.08
21 August	12.87	18.44	13.44	18.90	6.28	7.94	6.23	8.01

Note: The unit of soil moisture content is volume moisture percentage (%).

**Table 2:** Temperature settings at the experimental site.

Growth Stage	Maximum Temperature (°C)	Minimum Temperature (°C)
Germination stage	27	10
Seedling to ear stage	35	12
Flowering stage	35	15
Pollination to ripening	30	18

**Table 3:** Changes in maize yield and related traits under different treatments.

Treatment	BY (t/hm^2^)	EY (t/hm^2^)	EPN (1000 per/hm^2^)	GW (g/Ear)	HGW (g/100 Grains)	KPS (Grains/Ear)
JN01	30.87 ± 3.31 a	17.27 ± 2.05 a	67.13 ± 0.40 a	219.97 ± 29.17 a	43.47 ± 1.93 a	571.73 ± 41.19 a
JD01	19.70 ± 4.01 b	9.74 ± 0.45 c	63.19 ± 0.69 c	131.02 ± 3.96 b	30.58 ± 1.13 c	561.47 ± 11.32 ab
JN03	21.86 ± 1.59 b	14.03 ± 0.70 b	66.44 ± 0.40 ab	182.40 ± 12.45 a	36.35 ± 1.16 b	460.00 ± 10.21 c
JD03	20.49 ± 1.29 b	11.48 ± 0.65 b	65.74 ± 0.40 b	181.10 ± 20.38 a	32.92 ± 2.55 bc	510.93 ± 11.71 bc
*F*	8.546 *	24.199 **	36.667 **	15.360 **	29.439 **	17.561 **
*p*	0.007	<0.001	<0.001	0.001	<0.001	0.001

Note: Data are presented as mean ± standard error. Different lowercase letters within the same column indicate significant differences among treatments at *p* < 0.05 (Tukey’s HSD post hoc test). * indicates significant differences at *p* < 0.05; ** indicates highly significant differences at *p* < 0.01.

**Table 4:** Changes in maize ears under drought stress during the ear stage.

Treatment	EL (cm)	ED (mm)	BTL (cm)	NRE (Rows)	NGR (Grains)	CW (g)	TR (mm)
JN01	19.94 ± 0.77 a	53.06 ± 0.82 a	1.36 ± 0.13 a	14.3 ± 0.2 b	40.0 ± 3.1 a	38.94 ± 8.52 a	28.16 ± 0.45 a
JD01	14.78 ± 0.33 bc	46.50 ± 0.85 c	1.28 ± 0.10 a	14.0 ± 0.4 b	32.7 ± 1.3 b	38.7 ± 28.26 a	25.86 ± 0.56 b
JN03	15.14 ± 0.16 b	50.09 ± 0.21 b	0.58 ± 0.05 b	16.1 ± 0.2 a	34.9 ± 1.3 b	21.59 ± 0.39 a	25.76 ± 0.30 b
JD03	13.98 ± 0.15 c	48.59 ± 0.84 b	0.74 ± 0.12 b	15.7 ± 0.6 a	32.3 ± 0.8 b	20.76 ± 1.49 a	25.32 ± 0.12 b
*F*	116.786 **	42.449 **	42.327 **	22.222 **	10.969 *	1.427	31.638 **
*p*	<0.001	<0.001	<0.001	<0.001	0.003	0.305	<0.001

Note: Data are presented as mean ± standard error. Different lowercase letters within the same column indicate significant differences among treatments at *p* < 0.05 (Tukey’s HSD post hoc test). * indicates significant differences at *p* < 0.05; ** indicates highly significant differences at *p* < 0.01.

**Table 5:** Physiological and biochemical indicators of maize.

Character	Treatment	A	B	C
SOD	JN01	186.21 ± 30.73 a	161.95 ± 14.48 a	116.82 ± 13.14 ab
	JD01	219.41 ± 38.75 a	118.99 ± 12.18 a	75.20 ± 6.69 c
	JN03	192.02 ± 39.27 a	169.35 ± 33.07 a	135.02 ± 16.77 a
	JD03	214.50 ± 35.53 a	154.06 ± 22.41 a	88.96 ± 9.02 bc
POD	JN01	244.96 ± 18.32 a	273.21 ± 8.33 a	243.28 ± 4.76 a
	JD01	232.26 ± 15.46 a	240.24 ± 19.01 a	164.79 ± 8.22 c
	JN03	232.82 ± 17.32 a	277.29 ± 28.91 a	236.55 ± 11.45 a
	JD03	242.63 ± 18.17 a	251.14 ± 15.76 a	193.99 ± 9.97 b
CAT	JN01	135.56 ± 20.84 a	155.69 ± 8.92 a	98.57 ± 21.20 a
	JD01	128.44 ± 23.32 a	81.31 ± 17.42 b	73.19 ± 13.91 a
	JN03	137.94 ± 22.20 a	116.28 ± 22.91 ab	105.58 ± 16.20 a
	JD03	153.22 ± 11.49 a	116.46 ± 8.08 ab	99.21 ± 33.34 a
MDA	JN01	80.13 ± 8.06 a	90.94 ± 1.11 c	115.64 ± 1.69 b
	JD01	93.50 ± 6.60 a	139.77 ± 3.05 a	166.42 ± 16.02 a
	JN03	39.27 ± 2.08 b	62.21 ± 1.85 d	66.00 ± 3.34 c
	JD03	45.74 ± 4.33 b	105.34 ± 7.58 b	111.33 ± 13.07 b
Pro	JN01	13.93 ± 2.91 a	37.21 ± 2.86 c	64.39 ± 6.81 c
	JD01	11.39 ± 5.71 a	55.56 ± 6.27 b	250.92 ± 10.92 b
	JN03	13.76 ± 3.05 a	18.31 ± 3.18 d	80.76 ± 3.59 c
	JD03	10.02 ± 3.66 a	77.09 ± 7.40 a	356.68 ± 20.87 a

Note: Data are presented as mean ± standard error. Different lowercase letters within the same column indicate significant differences among treatments at *p* < 0.05 (Tukey’s HSD post hoc test).

## Data Availability

All the sequencing data that support the findings of this study are openly available in NCBI, BioProject accession number PRJNA1483246, https://www.ncbi.nlm.nih.gov/sra/PRJNA1483246 (accessed on 7 July 2026).

## Abbreviations

The following abbreviations are used in this manuscript:

EY	Economic yield
BY	Biomass yield
EL	Ear length
ED	Ear diameter
EPN	Effective panicle number
GW	Grain weight per spike
CW	Cob weight
TR	Thick Rachis
BTL	Bald tip length
NRE	Number of rows per ear
NGR	Number of grains per row
HGW	Hundred-grain weight
KPS	kernels per ear
